# Detecting Unknown Attacks in Wireless Sensor Networks That Contain Mobile Nodes

**DOI:** 10.3390/s120810834

**Published:** 2012-08-07

**Authors:** Zorana Banković, David Fraga, José M. Moya, Juan Carlos Vallejo

**Affiliations:** Departamento de Ingeniería Electrónica, ETSI Telecomunicación, Universidad Politécnica de Madrid, Av. Complutense, 30, 28040 Madrid, Spain; E-Mails: dfraga@die.upm.es (D.F.); josem@die.upm.es (J.M.M.); jcvallejo@die.upm.es (J.C.V.)

**Keywords:** wireless sensor networks, mobility, unknown attacks, clustering algorithms, reputation systems

## Abstract

As wireless sensor networks are usually deployed in unattended areas, security policies cannot be updated in a timely fashion upon identification of new attacks. This gives enough time for attackers to cause significant damage. Thus, it is of great importance to provide protection from unknown attacks. However, existing solutions are mostly concentrated on known attacks. On the other hand, mobility can make the sensor network more resilient to failures, reactive to events, and able to support disparate missions with a common set of sensors, yet the problem of security becomes more complicated. In order to address the issue of security in networks with mobile nodes, we propose a machine learning solution for anomaly detection along with the feature extraction process that tries to detect temporal and spatial inconsistencies in the sequences of sensed values and the routing paths used to forward these values to the base station. We also propose a special way to treat mobile nodes, which is the main novelty of this work. The data produced in the presence of an attacker are treated as outliers, and detected using clustering techniques. These techniques are further coupled with a reputation system, in this way isolating compromised nodes in timely fashion. The proposal exhibits good performances at detecting and confining previously unseen attacks, including the cases when mobile nodes are compromised.

## 1. Introduction

The development of Wireless Sensor Networks (WSNs) was mainly motivated by military applications, such as control and surveillance in battlefields, but over time their deployment has been introduced to other areas, *i.e.*, industrial control and monitoring, environmental monitoring, health monitoring of patients or assistance to disabled people and the emerging field of ambient intelligence. In all of the applications, it is mandatory to maintain the integrity and the correct operation of the deployed network. Furthermore, WSNs are often deployed in unattended or even hostile environments, making their securing even more challenging. In addition, the trends in the recent past are to include mobile nodes, since this can make the WSN more resilient to failures, reactive to events, provide better coverage of the monitored area, and able to support disparate missions with a common set of sensors. However, mobility additionally complicates the security issue.

WSNs consist of huge numbers of sensor nodes, and since this number is huge, the nodes have to be very cheap. This further implies that they possess very limited power and computation resources, small memory size and limited bandwidth usage. Furthermore, the incorporation of any tamper-resistant hardware would assume unacceptable costs. All of this makes the security of these networks very challenging, as the resource limited devices cannot support the execution of any complicated algorithms. Moreover, WSNs use a radio band that is license-free, so anybody with appropriate equipment can listen to the communication. Finally, due to their deployment in areas that are difficult to reach makes them prone to node failures and adversaries.

On the other hand, profound analysis of the state of the art has allowed us to identify the main issues of the existing solutions, which are their limited scope of detection, as the majority of them can detect only previously seen attacks, and the fact that any adjustment has to done by humans, which cannot be done in a timely fashion due to the deployment of the nodes in hard to reach areas. In order to overcome these issues, we have proposed an approach based on anomaly detection that is able to detect a wide range of attacks, including the previously unseen ones, without the necessity to have any previous knowledge on the attacks and their way of operating. Attacks are treated as data outliers, and since outliers are defined as something different from the normal, we can classify our approach as an anomaly detection one. Thus, the basic premise of this approach is that the attacks are deviations from normality. However, not all deviations from normality are attacks, but we believe that they have to be reported and examined further. For this reason, in this work we only provide a first reaction to anomalies, which is their isolation, but it is assumed that the base station has additional technique to decide whether an anomaly can be attributed to an attack, or not. However, this is out of the scope of this work.

The existing anomaly detection solutions mainly look for the deviations in the values of the parameters that capture the properties of known attacks, which means that they use so-called numerical features. Hence, their possibilities to detect unknown attacks are limited, since it is hard to define the numerical features of the unknown attacks. In order to overcome this issue, we have proposed a machine learning solution for anomaly detection along with the feature extraction process that does not capture the properties of the attacks, but rather relies on the existing temporal and spatial redundancy in sensor networks and tries to detect temporal and spatial inconsistencies in the sequences of sensed values and the routing paths used to forward these values to the base station. In this work we further propose a special way to integrate mobile nodes into this approach, given that mobility is a big issue in anomaly detection, as it can lead to observation data that have long range dependency and in this way increase its difficulty. Moreover, it is greatly unexplored and untreated subject in the current state of the art.

The data produced in the presence of an attacker are treated as outliers, and they are detected using clustering techniques. The techniques are further coupled with the reputation system, which provides implicit response to the attackers, as the compromised nodes get isolated from the network. The proposal has been tested in the presence of the attacks that were unknown during the training, exhibiting good performances at detecting and confining these attacks. To summarize, the objective of this work is to detect unknown attacks in WSNs that contain both static and mobile sensor nodes, providing special treatment for the latter ones given their dynamic nature, and provide initial response to malicious nodes. The main contribution of this work is the proposal for special treatment of mobile nodes, which provides their efficient incorporation in the existing approach, while maintaining high performance level.

The rest of the work is organized as follows: Section 2 gives more details of the state of the art solutions. Section 3 details the proposed solution, while Section 4 provides its evaluation. Finally, conclusions are drawn in Section 5.

## 2. Related Work

A number of custom IDSs for sensor networks have been proposed. Some representative solutions are given in [1,2]. However, they are mainly focused on misbehaviour detection, and hence are capable of detecting only a limited number of attacks, *i.e.*, known attacks and their variations. In order to detect new attacks, they need to be adjusted by humans.

Recently a few solutions that deploy machine learning techniques have appeared [3,4]. Among these solutions we can also find a few anomaly-based solutions [5,6], that claim to have the capability to detect unknown attacks. They uphold the idea that machine learning techniques offer higher level of flexibility and adaptability to the changes of the environment, as it only requires retraining the algorithms with new data, which can be done automatically. Furthermore, in reality we often have to deal with incomplete and noisy information, and the security requirements themselves are often fuzzy and incomplete. Machine learning techniques are known to cope well with these sorts of problems, which is the main reason they are becoming part of security solutions, even commercial ones [7].

However, the feature sets they deploy mostly include those features that capture the properties of known attacks, *i.e.*, those that are known to change under the influence of an attacker, or are known to be weak spots. This is their major deficiency, as relying on these features only known attacks or their variations can be detected. Furthermore, it assumes that an attacker can exploit only the known vulnerabilities, but general experience is that vulnerabilities are detected after being exploited by an adversary. Some of them assume that the feature sets can be expanded [1], yet this again has to be done through a human intervention. In addition, taking into consideration the existence of mobile nodes in the network is greatly unexplored and untreated subject in the current state of the art [8]. A summary of the most representative existing solutions for intrusion detection is given in Table 1, from which we can conclude the following: although there are many proposed solutions, none of them is general enough to be able to handle a greater variety of attacks. For this reason, most of the solutions should work aside with few more that address different aspects of security breaches. However, this can introduce high overhead and consume significant resources. Another issue is that most of them are able to detect known attacks, but the experience from the network security tells us that the attackers always manage to find new possibilities to launch their attacks. Finally, almost none of them address difficulties introduced by mobile nodes. One example is given in [6], but it just deals with the attacks on routing protocols. Furthermore, this work does not assume any special treatment of mobile nodes. Thus, our aim is to provide a machine learning based solution that does not suffer from these issues, *i.e.*, a solution that would be capable of detecting wide range of attacks, including the previously unseen ones, which would also be adaptable automatically and address the existence of mobile nodes in the network.

## 3. Proposed Solution

### 3.1. Envisioned WSN Model

**Figure 1.**
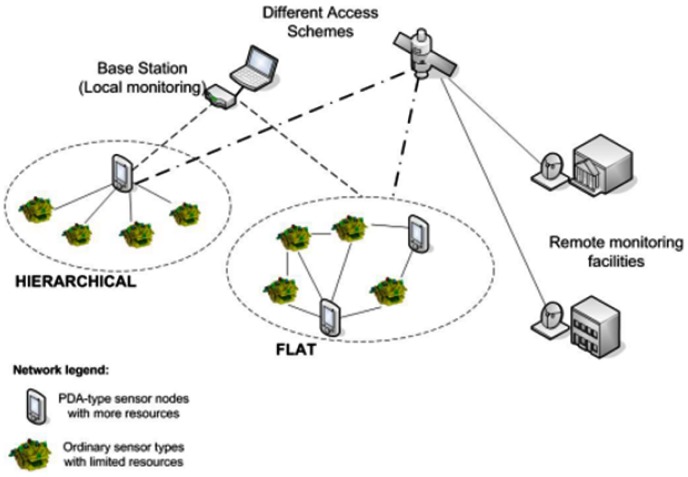
Envisioned WSN Model.

We envision wireless sensor networks (Figure 1) where most of the sensor nodes exhibit limited resources, but there are also a number of PDA-like sensors with more computational resources, memory and battery capacity. There is at least one base station as well. Their number is significantly smaller than the number of the “normal” sensors, usually a few orders of magnitude smaller. The nodes can organize themselves either in a hierarchical or flat manner. Nodes can be fixed or mobile, although it is assumed that the majority of the nodes are fixed. No constrains regarding routing protocol are assumed.

### 3.2. Attack Assumptions

In order to provide uninterrupted network operation, core network protocols (aggregation, routing and time synchronization) have to be secured. Regarding the attacks on the aggregation protocol [10], we assume that they demonstrate themselves as skewed aggregated values, which can be the result of either a number of skewed sensed values, or a compromised aggregated node. The assumption is very reasonable, bearing in mind that the main objective of these attacks is to provide a wrong picture of the observed phenomenon, or wrong context information in context aware systems. On the other hand, in time critical systems it is mandatory to receive information within a certain time window. If the attacker manages to introduce delays or desynchronize clock signal in various nodes, the received critical information will not be up to date, which can destabilize the system. Also, if the received information is not up to date, the aggregated value will be skewed, as it will also be out of date. For these reasons, and given the existing redundancy in WSNs, we believe that these attacks can be detected as temporal and/or spatial inconsistencies of sensed values.

Regarding attacks on routing protocols [10], we assume that they will introduce new and different paths than those that have been seen before. Here we have attacks whose main objective is to compromise the routing protocol, so the data do not reach the sink, or the sink is reached with a considerable delay, which makes the data useless. These attacks usually spoof or alter the data stored in the routing tables of the nodes. Thus, the resulting routing paths will be different from those used in a normal situation. The other group of attacks, which are more dangerous, has the objective of tampering with the data, and they usually do it in two steps: in the first step they establish themselves as an attractive routing hop (e.g., by falsely presenting lower cost of its routes), so the majority of the data goes through that node, and in the second step they tamper with the data. In the case of wormholes for example, two nodes that are not within each other's radio range result in consecutive routing hops in routing paths, which is not possible in a normal situation. From these examples we can see that the assumption about the attacks resulting in routing paths different from those that appear in normal situation is reasonable. Thus, in this case we want to detect temporal inconsistencies in paths used by each node.

The attacks can be either mote-based or laptop-based, but can be either inside or outside based. Another assumption is that the attack always starts after the initialization of the network, *i.e.*, the network functions normally for some time, which is a very reasonable assumption.

### 3.3. Feature Extraction and Formation of Model

Our idea is to find temporal and/or spatial inconsistencies in sensed data in order to detect manipulated data and/or compromised nodes. For this reason, we follow the idea presented in [11,12], based on extracted n-grams and their frequencies within different time windows. Thus, the vectors used for characterization that allow the deployment of machine learning are composed of the extracted n-grams. For the purpose of illustration, we will give a short example for a sensor that detects presence. Let a sensor give the following output during the time window of size 20: 1 1 1 1 0 0 0 0 0 0 1 1 1 1 1 1 0 0 0 0. If we fix the n-gram size on 3, we extract all the sequences of size 3 each time moving one position forward. In this way we can observe the following sequences and the number of their occurrences within the time window: 111—occurs six times, 110—two, 100—two, 000—six, 001—one, 011—one. Thus, the extracted vector that corresponds to this time window contains the following features: 111—0.33, 110—0.11, 100—0.11, 000—0.33, 001—0.06, 011—0.06. In our model, the sequences are the features and their frequencies are the corresponding feature values. Thus, the sum of the feature values is always equal to 1. In our algorithm this characterization, *i.e.*, the extraction of vectors, is performed at predefined moments of time and takes the established amount of previous data, e.g., we can perform the characterization after every 20 time periods based on the previous 40 values.

In a similar fashion, we form features for spatial characterization. The first step is to establish vicinities of nodes that historically have been giving consistent information. Furthermore, since an agent is supposed to reside on a node, vicinities are established using the nodes whose information can reach the agent. In this way, an n-gram for spatial characterization in a moment of time is made of the sensor outputs from that very moment. For example, if sensors S1, S2, S3 that belong to the same group each give the following output: 1 1 1 0 during four time epochs, we characterize them with the following set of n-grams (each n-gram contains at the first position the value of S1, the value of S2 at the second and the value of S3 at the third at a certain time epoch): 111—occurs three times, 000—occurs once, thus the feature value of each n-gram is: 111—0.75, 000—0.25, *i.e.*, the frequencies within the observed period of time.

We develop the same principle for characterizing routes that a node has been using to send its sensed data to the sink. Each routing hop adds its ID to the message that is further forwarded, so the sink has the information about the routing path together with the message. However, this is not performed with each message in order to avoid the overhead in the communication channel. Yet, considering that one routing path is usually used more than once, it is reasonable to assume that the sink will have all the paths used for routing the data from a certain sensor. As previously mentioned, each sensor has its own model and each feature, *i.e.*, n-gram in the model consists of a predefined number of successive hops used in routing information coming from the node. For example, if during the characterization time, the node has used the following paths for routing its data to the sink: A-B-C-S—three times, A-D-E-F-S—two times, A-B-E-F-S—one time (A—the node that is sending the data, B, C, … —other nodes in the network, S—sink), we can characterize the routing with the following n-grams (n = 3): ABC, BCS, ADE, DEF, EFS, ABE and BEF. In all of the routes, the n-gram ABC occurs three times, BCS—three, ADE—two, DEF—two, EFS—three, ABE—one, BEF—one. The total number of n-grams is 15, so dividing the values given above with 15, we get the frequencies of each n-gram which are the values that we assign to our features, *i.e.*, n-grams.

### 3.4. Deployed Distance Function

Since some of the n-grams can appear more than once, it is obvious that the extracted vectors will not be of constant size. Thus, we cannot use standard distance functions. The distance between the instances of the presented model is taken from [13]. It is designed to calculate the distance between two sequences. We have elected this one (among all those given in [13]) since it is proven to be the most efficient in the terms of the absolute execution time.

### 3.5. Scope of Attacks Covered with the Approach

As previously mentioned, we treat attacks as data outliers and deploy clustering techniques, namely SOM, unsupervised GA and Growing Neural Gas (GNG). Further details on the algorithm implementation can be found in [12,14,15], while their distributed organization will be described in more detail in Section 3.7. In the following we will explain the principles of the approach. It is important to mention here that the algorithms can be trained with both clean and “unclean” data (contains traces of attacks). Furthermore, the algorithms are constantly retrained in order to decrease time lags between model training and model application. The retraining frequency depends on the dynamics of the underlying sensor network.

There are two approaches for detecting outliers using clustering techniques [16] depending on the following two possibilities: detecting outlying clusters or detecting outlying data that belong to non-outlying clusters. For the first case, we calculate the average distance of each cluster to the rest of the clusters (or its closest neighborhood) (*MD*). In the latter case, we calculate quantization error (*QE*) of each input as the distance from its corresponding cluster center.

The attacks that can be detected with the proposed approach are those that introduce changes into either the sensed value that is forwarded to the base station or the routing paths. These changes will result in different distribution of the extracted n-grams. However, if we take frequencies as feature values, the sum of the feature values remain the same, *i.e.*, 1, so we can write the following equation:

(1) $$\sum_{i=0}^{N}\Delta f_{i}=0$$

where N is the total number of the extracted n-grams and *Δf_i_* is the change of the feature value of the n-gram *i*. On the other hand, according to the distance function [13], the introduced change in distance between the attacked instance and any other is:

(2) $$AD=\sum_{i=1}^{N}\left|\Delta f_{i}\right|$$

In essence, this is the change introduced in the above defined *QE* or/and *MD* values. Thus, the following inequality defines the changes introduced by the attacks:

(3) $$\sum_{i=1}^{N}\left|\Delta f_{i}\right|>f_{th}$$

where *f_th_* is the threshold value used to distinguish attacks from normal situations.

Now we will see how the changes introduced by the attacker affect the feature values. Bearing in mind that each sensed value or routing hop participates in n features, where n is the size of the n-gram, if the attacker changes one value, the values of *2n* (at most) features will be changed [the values of newly created n-grams (n at most) with the change will increase, while the values of those that existed before the change (again n at most) will decrease]. For example, the third element in the sequence …1 0 0 1 1… for n = 3 participates in three n-grams: 100, 001 and 011. However, if the attacker changes this value into 1, the sequence becomes …1 0 1 1 1…, in which case the third element participates in these n-grams: 101, 011 and 111. This results in decreased occurrences of the n-grams 100 and 001, while the occurrences of the 101 and 011 become increased (011 appears in both cases, so its total occurrence remains the same). In total, the occurrence of four n-grams is changed. For these reasons, if the attacker introduces *N_err_* change in the sample of the size *N_sample_*, the value of *ΔD* will range between 0 (in the case the changes are symmetric, so the effect of one change cancels the effect of another and the distribution does not change at the end), and the value that corresponds to the case when the effects of each change are completely uncorrelated, so they sum together, which is given with the following formula:

(4) $$D_{\max}=2n*f_{\text{err}}=\frac{2nN_{\text{err}}}{N_{\text{sample}}}$$

Thus, having in mind the correlation of the n-grams, in order to model this change that ranges from 0 to *D_max_* we use the next formula:

(5) $$F\left(\rho \right)=\beta +\left(1-\beta \right)e^{k\alpha }$$

where *α* = 1 – 1/*ρ*, *β*(<1, since the function should grow with *ρ*) and *k* are constants defined in the design process (the specific meaning of both will be explained later in this section) and *ρ* is the coefficient of total correlation between the n-grams. The value of *F*(*ρ*) is *β* for *ρ* = 0, (the reason for this will be explained in the following), and 1 for *ρ* = 1.

The coefficient of total correlation [17] expresses the amount of dependency that exists among a set of variables. For a given set of k random variables *X_1_, X_2_*, …, *X_k_*, the total correlation *C*(*X_1_, X_2_*, …, *X_k_*) is given by the following formula:

(6) $$C\left(X_{1},X_{2},\ldots ,X_{k}\right)=\sum_{i=1}^{k}H\left(X_{i}\right)-H\left(X_{1},X_{2},\ldots ,X_{k}\right)$$

where *H*(*X_i_*) is the information entropy of variable *X_i_*, while *H*(*X_1_*, *X_2_*, …, *X_k_*) is the joint entropy of the variable set {*X_1_*, *X_2_*, …, *X_k_*}. In our case, the variables are the extracted n-grams. For the sake of calculating the above formula, their distribution can be approximated either as a common distribution depending on the purpose of the deployed sensor network, or using the historical data sensed by the network.

Regarding the value of *β*, we have to take into account that the higher the value of *β* is, the function becomes closer to its asymptotic function *F*(*ρ*) = 1. Thus, the effect of *ρ* becomes smaller. Similar stands for the value of *k*. As k → 0, the function becomes closer to the same asymptotic function. In the opposite case, as *k* → ∞, the function reaches its asymptote: *F*(*ρ*) = 0 for *ρ* < 1, *F*(*ρ*) = 1 for *ρ* = 1. In both cases the effect of *ρ* becomes less significant.

Finally, we get the following formula:

(7) $$F\left(\rho \right)\frac{2nN_{\text{err}}}{N_{\text{sample}}}>f_{th}$$

which gives us the minimal number of changes the attacker has to introduce in order to be detected by the approach:

(8) $$N_{\text{err}\min}=\frac{N_{\text{sample}}}{2nF\left(\rho \right)}f_{th}$$

In the previous equation we have the following degrees of freedom: *N_sample_*, n and *f_th_*. Lower characterization periods (*N_sample_*) and the threshold on one side and higher n on the other give us the opportunity to detect the attacker even if he introduces very few changes. However, this can also result in higher false positive rate, so a tradeoff between higher detection and lower false positive rate has to be established. This depends on many factors, such as the application of the deployed WSN or the existing redundancy. Also, the values of both *β* and *k* indirectly affect on this value through *F*(*ρ*). As the value of *β* increases or the value of *k* decreases, the value of *F*(*ρ*) for the same *ρ* increases, which further decreases the value of *N_errmin_*. In opposite cases, as the value of *β* decreases or the value of *k* increases, the value *N_errmin_* will increase.

The previous formula also helps us to define the minimal value of *β*. It derives form the constraint that the maximal possible value of *N_errmin_* is equal to *N_sample_*. For the same reason, *F*(*ρ*) has to be different than 0 for *ρ* = 0 (in the opposite case, *N_errmin_* → ∞). This results in following:

(9) $$\beta >\frac{N_{\text{sample}}}{2nN_{\text{err}\min}}f_{th}$$

### 3.6. Recovery from Attacks

Every sensor node is being examined by agents that execute one of the algorithms for detecting attacks, which reside on nodes in its vicinity and listen to its communication. The agents are trained separately. The system of agents is coupled with a reputation system [18] where each node has its reputation value that basically reflects the level of confidence that others have in it based on its previous behavior. In our proposal, the output of an agent affects the reputation system in such a way that it assigns lower reputation to the nodes where it detects abnormal activities and *vice versa*. We further advocate avoiding any kind of interaction with the low-reputation nodes: to discard any data or request coming from these nodes or to avoid taking them as a routing hop. In this way, compromised nodes remain isolated from the network and have no role in its further performance. After this, additional actions can be performed by the base station, e.g., it can revoke the keys from the compromised nodes, reprogram them, *etc*.

In this work the reputation is calculated in the following way: *f_th_* is taken to be 1 for the following reasons. Considering that the attacks will often result in the creation of new n-grams, it is reasonable to assume that the extracted vector in the presence of attackers will not be a subset of any vector extracted in normal situation, thus the distance will never be lower than 1. We further define two reputation values, *repQE* and *repMD* based on the previously defined *QE* and *MD* values and afterwards joint reputation rep used for updating overall reputation based on these two values:

if (QE < 1) { repQE = 1; } 
else { repQE = 1 - QE/2; }
if (MD < 1) { repMD = 1; } 
else { repMD = 1 - MD/2; }

The value (rep) for updating overall reputation is calculated in the following way:

if (QE > 1) { rep = repQE; } 
else { rep = repMD; }

There are two functions for updating the overall reputation of the node, depending whether the current reputation is below or above the established threshold that distinguishes normal and anomalous behavior. If the current reputation is above the threshold and the node starts behaving suspiciously, its reputation will fall quickly. On the other hand, if the reputation is lower than the established threshold, and the node starts behaving properly, it will need to behave properly for some time until it reaches the threshold in order to “redeem” itself. The first objective is provided by the function x + log(1.2x). Finally, the reputation is updated in the following way:

if (last_rep[node] > threshold) {
new_rep[node] = last_rep[node] + rep + log(1.2 * rep); }
else {
new_rep[node] = last_rep[node] + c_limit * (rep + log(1.2 * rep)); }

The second objective is provided by the coefficient *c_limit*, which takes values lower than 1 and its purpose is to limit selective behavior of a node by decreasing the reputation growth if the reputation value is below the threshold. Very low values of this coefficient obligate nodes to behave properly most of time. If the final reputation value falls out from the [0, 1] range, it is rounded to 0 if it is lower than 0 or to 1 in the opposite case. The threshold value can be set to the middle of the reputation value range (50 in our case) at the starting point. However, this value depends on many different factors. One of the most important factors is risk, and the threshold value is proportional to it: if the operation in the network (or in some of its parts) is critical, the threshold value should be higher, and *vice versa*. Thus, a process that evaluates risk should be able to update the threshold value.

However, if during the testing of temporal coherence, we get normal data different from those that the clustering algorithms saw during the training, it is possible to get high *QE* value as well. On the other hand, the spatial coherence should not detect any anomalies. Thus, the final reputation will fall only if both spatial and temporal algorithms detect anomalies. In the opposite case, its reputation will not change significantly. This is implemented in the following way:

if (value_rep < threshold) { 
if (space_rep < threshold) {  
result = value_rep; 
} else { result = 1 - value_rep; }
} else { 
result = value_rep; }where value rep is the reputation assigned by the algorithms for temporal characterization and space rep is the reputation assigned by the algorithms for spatial characterization. On the other hand, as mentioned in the previous text, in the situations such as the data coming from a node exhibits large variations, temporal inconsistencies are not likely to be detected. However, spatial inconsistencies are very likely to be detected. Thus, spatial inconsistence is sufficient in order to raise an alarm.

Concerning the detection of routing protocol anomalies, the explained approach can tell us if there is something suspicious in routing paths of a certain node. Yet, in order to find out the nodes that are the origin of the attack, we need to add one more step. In this second step, if the reputation of the routes calculated in the previous step is lower than the established threshold, the hops that participated in the bad routes will be added to the global list of bad nodes, or if they already exist, the number of their appearance in bad routes is increased. The similar principle is performed for the correct nodes. For each node, let the number of its appearances in bad routes be *nBad* and the number of its appearances in good routes be *nGood*. Finally, if *nGood* is greater than *nBad*, the node keeps its reputation value, and in the opposite case, it is assigned the following reputation value:

(10) $$\frac{\text{nGood}}{\text{nGood}+\text{nBad}}$$

In this way, as the bad node spreads its malicious behavior, its reputation will gradually decrease.

### 3.7. Distributed Organization of Detectors

Given the distributed nature of WSNs, the detection should be organized in a distributed manner as well. In our approach detectors are implemented as software agents and they reside on physical nodes. It is important to notice that machine learning techniques have many parameters that should be set from the start, e.g., duration of training, size of the lattice in the case of SOM, crossover and mutation probabilities in the case of GA, *etc*. It is not easy to guess the optimal parameters *a priori*, and in our case an additional problem is the impossibility of human interaction. Moreover, in the case where an agent resides on a compromised node, it is possible for the attacker to compromise the agent as well. We consider that additional security measures that protect the agent from the host (and *vice versa*) are taken, such as those proposed in [19], so agent subversion is not a straightforward process. The detailed explanation of these techniques is out of the scope of this work.

In order to overcome these issues, we introduce agent redundancy, where more than one agent monitors the same node. Each physical node may contain more than one agent. In the beginning we have a group of agents that implement one of the proposed algorithms with different parameter settings. Every node is being examined by an agent that resides on another node in its vicinity and which promiscuously listens to its communication. Each of the agents is trained separately. Final decision can be made either applying majority voting, or a weighted sum, where each weight depends on the “quality” of each agent. A simple and efficient way of calculating this quality could be to introduce agent reputation (in this way we would have a hierarchical reputation system, where the agents would assign reputation to the nodes, while there will also exist a reputation system implemented in the base station for the agents as well). This reputation can be calculated using beta function [20], which has strong background in the statistics theory:

(11) $$R=E\left(\text{Beta}\left(\alpha +1,\beta +1\right)\right)=\frac{\alpha +1}{\alpha +\beta +1}$$

where *α* stands for the number of correct decisions made by the detector, while *β* stands for the number of incorrect ones. The voting system decides whether a response is right or wrong based on majority voting.

### 3.8. Incorporation of Mobile Nodes

Mobile nodes introduce additional alterations in the system, making the detection process more complicating. However, the presence of mobile nodes should not affect the proper functioning of the WSN, *i.e.*, they should be able to sense the environment, continue sending their own sensed values and be used as a routing hop in forwarding sensed values of other nodes as well. Due to their special nature, these nodes require special treatment in the proposed detection system.

In order to minimize the disruption of the detection system, we propose the following principle: if the nodes encounter with a new node in their area, they will ask the base station about its reputation and continue the interaction assuming the existing reputation value. Concerning a mobile node that has changed the position, we distinguish two possible situations: the node remains in the same area, *i.e.*, it has not changed its position significantly, which can be concluded by the base station if it still uses similar group of nodes to route its data, or if it starts using completely new routes, the old model has to be discarded and a new model has to be established and trained for the same node. This goes for both models: the one based on sensed values, *i.e.*, if a node has changed its position significantly, it is very probable that sensed values will be different, thus a model has to be established by performing the training with new data, and also the one based on routing information.

Regarding the nodes whose routes will be changed with the introduction of a new node that behaves properly, according to the presented model and the adopted distance function, it is not possible to introduce significant change so as to raise doubts, thus it is not necessary to perform the re-training. However, if more new nodes appear in the routes, it is advisable to perform the re-training (with both old and new data) in order to avoid false positives.

## 4. Experimental Evaluation

### 4.1. Simulation Environment

The proposed approach has been tested on a simulator of sensor networks developed by our research group and designed using the C++ programming language. We have decided to design a simulator mainly because there is no available testbed for security applications in sensor networks. Attacking recorded data from a testbed does not significantly differ from the simulation. What's more, the available testbeds for wireless sensor networks contain a relatively small number of sensors (100 at most), in which case the data obtained from our simulator are more complex (simply because there are more sensors). For these reasons, we believe that until a testbed for security applications in WSNs appears, a simulator is a better choice for testing security applications. We further evaluated two well-known WSN simulators, ns-2 [21] and Castalia [22] over Omnet++ [23]. Yet, we eventually decided to implement our own simulator, TRSSim. This simulator implements only the basic functionality of the communication layers, rather than implementing all layers in detail, which anyhow is not very important for our application. This significantly reduces the total simulation time, which was our main reason for implementing the simulator. In the following we will describe the characteristics of the simulator that are pertinent for understanding the results of this work. The experiments, however, do not rely on any TRSSim-specific data or constraint and they can be replicated easily with other simulators.

The network is organized as clusters of close sensors where each group has its cluster head, as is often done in real networks in order to reduce computational overhead and energy consumption. Cluster heads are the only sensors that can participate in the communication between different clusters and also in routing. The mobility model is random-based, *i.e.*, nodes can move randomly in any direction towards a random destination, with the restriction of the maximal distance between the current position and the destination. The maximal distance in our case is 20% of the distance between the current node position and the base station in order to avoid the situation where the node ends up at the position of the base station.

In this work we will present the results based on the Sybil attacks [10], where the malicious node pretends to have multiple IDs, either false, *i.e.*, fabricated, or impersonated from other legitimate nodes, *i.e.*, stolen IDs. Added malicious nodes send random values that may or may not coincide with the values sent by the original good nodes. Since in this work we are dealing with unknown attacks, clustering algorithms are trained with data that have no trace of attacks. The performance of the approach when attacks are present during the training can be found in our previous work [11,14,16]. Thus, we can say that the attack is unknown to the algorithm. In our work both Sybil nodes and the compromised nodes whose IDs have been stolen are treated as malicious. Although the compromised nodes do not perform any malicious activity, we believe they should remain isolated until the security response system deals with the attack, e.g., until the base station changes their ID.

The proposed algorithm has been tested on the presented simulated sensor network that contains 40 sensor nodes that can be placed in 100 different positions. The network simulates a sensor network for detecting presence in the area of application. The groups for spatial characterization are formed in the following way: close sensors that should give the same output are placed in the same group.

The duration of the experiment is 1000 time ticks. One time tick in simulator is the period of time required to perform the necessary operations in the network, and it is equivalent to a sampling period, or time epoch in sensor networks. In the following we will present results in different scenarios varying the attack strength.

### 4.2. Results

**Figure 2.**
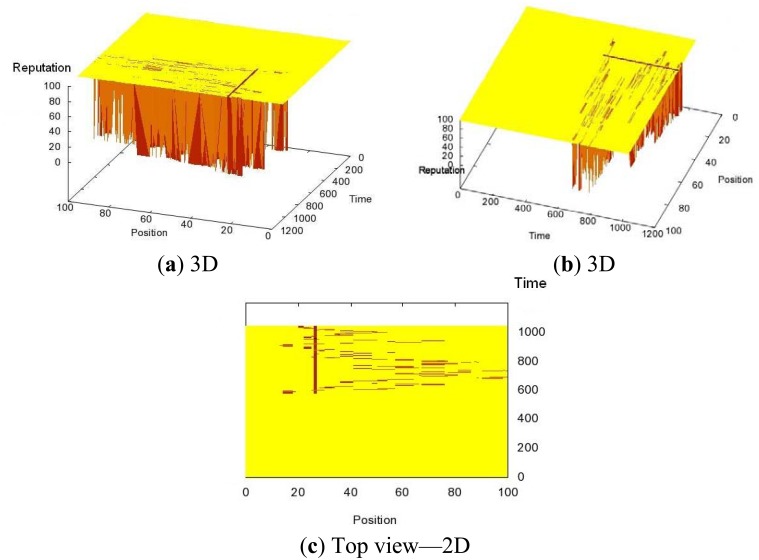
Reputation Evolution in Time and Space—Different Views.

In order to illustrate the performance of the algorithm, in the first experiment the Sybil node is static (it is added at the position 26) and it has the valid ID of a mobile node. In Figure 2(a,b), we can observe from different angles that the reputations of both the Sybil node at the position 26 and the compromised mobile node have been significantly lowered, which means that we have successfully detected and confined the attack. In Figure 2(c), we present the top view, where we can distinguish the path of the compromised mobile node due to its decreased reputation.

**Figure 3.**
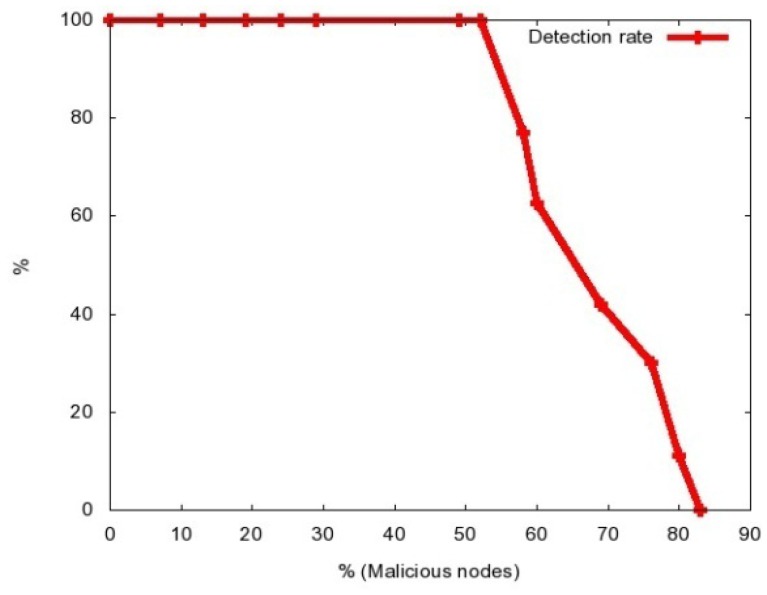
Detection Rate *vs.* % of Malicious Nodes.

In the following experiments we will gradually increase the number of compromised nodes, increasing as well the number of attacked mobile nodes. In Figure 3 we present the dependence of the average detection rate of the percentage of the malicious nodes.

As we can observe, with the proposed approach we can detect the attack if up to 80% of the nodes are malicious, and completely confine it (*i.e.*, achieve 100% detection rate) if up to 52% of the nodes are malicious. It is also important to mention than in all the experiments the false positive rate was 0%.

**Figure 4.**
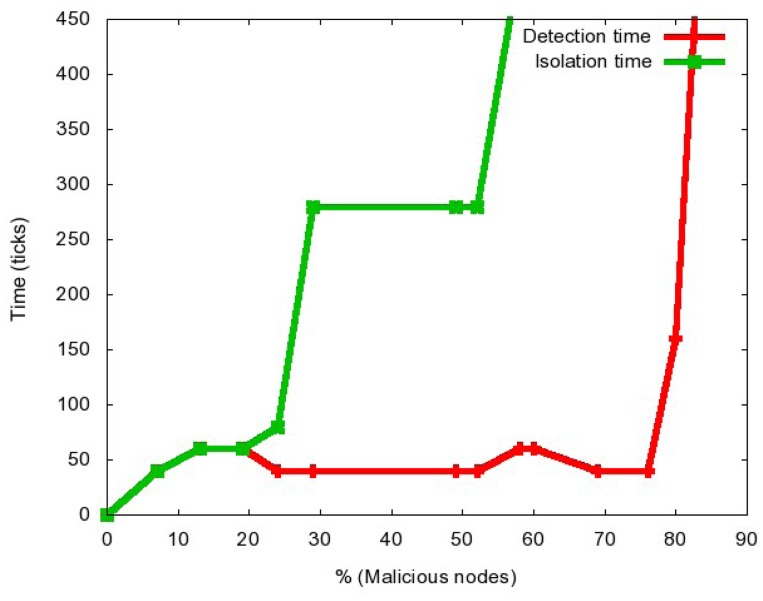
Detection and Isolation Time.

In Figure 4 we show how the time of detection and the complete isolation of the attack depend on the total number of malicious nodes in the network. As we can observe, the attack cannot be isolated if more than 58% of the nodes are malicious, nor it can be detected if more than 80% of the nodes are malicious. On the other hand, if up to 20% of the nodes are malicious, the attack is detected and confined in the same moment, which is due to the fact that the great majority of nodes are still behaving properly and it is not complicated to distinguish the misbehaving ones. As the attack becomes more aggressive, it is harder to detect and isolate all the misbehaving nodes.

## 5. Conclusions

In this work we have proposed a machine learning based anomaly detection approach for detecting unknown attacks in wireless sensor networks. We have also proposed a way to integrate mobile nodes in the approach, which is the main novelty of this work. The attacks are treated as data outliers, and we have designed clustering algorithms for outlier detection. The algorithms are further coupled with a reputation system, which provides implicit response to attackers, as low reputation nodes remain isolated from the network. Our experiments confirm that the approach is capable of detecting and completely confining attacks that were unknown to the algorithms during the training, with no false positives, and even in the cases mobile nodes have been attacked. We were able to achieve 100% detection rate of up to 52% of the nodes were malicious, and detect the presence of the attack if up to 80% of the nodes are malicious.

In the future we plan on broadening the scope of attacks our approach can detect by addressing attacks that compromise mobility patterns, which can make the approach helpful in detecting attacks in cellular networks or in detection of mobile intruders in the monitored area in surveillance applications [24].

## Figures and Tables

**Table 1.** Summary of the representative solutions.

			**Lidea** [1]	**SVM** [5]	**ML_FW** [9]	**IDRout** [6]	**H-IDS** [2]
**Classification**	Signature		✓		✓		✓
	Anomaly		✓	✓		✓	
**Attack Scope**	Limited		✓	✓	✓	✓	✓
	General						
	Unknown attacks		✓*			✓*	
**Adaptable**	Yes	Automatic		✓			
		Human Interaction	✓		✓	✓	✓
	No						
**Mobility**	Yes					✓	
	No		✓	✓	✓		✓

* Claimed, not proven.
